# Editorial: Dance and Disability

**DOI:** 10.3389/fpsyg.2022.878253

**Published:** 2022-03-16

**Authors:** Susanne Quinten, Bettina Bläsing, Sarah Whatley

**Affiliations:** ^1^Music and Movement in Rehabilitation and Education, Department of Rehabilitation Sciences, Technical University Dortmund, Dortmund, Germany; ^2^Centre for Dance Research (C-DaRE), Institute for Creative Cultures Building, Coventry University, Coventry, United Kingdom

**Keywords:** dance, disability, mixed-ability dance, inclusive dance, dance therapy, community dance, cultural participation

In the past decades, dance has developed into a research subject whose knowledge-generating potential is now recognized in many scientific disciplines, from the social sciences, education, cognitive and neuroscience, health and rehabilitation sciences to the arts. With growing need and motivation to provide for inclusive scenarios in all areas of life, mixed-ability dance, in which dancers with different bodily, sensory and mental conditions dance together and collaborate on equal terms, is giving a new, strong impetus to the scientific and artistic research landscape. Multidisciplinary research that considers methods and approaches as well as questions and ideas from all related fields has the potential to foster knowledge processes that challenge the normative and presumptions of what is “normal.” Importantly, it can also contribute to the cultural participation of people with disabilities and to the further development of inclusive education, in the arts and elsewhere.

With this Research Topic on “*Dance and Disability*,” the editors wish to emphasize and advance this development. The seven articles featured in this collection highlight approaches and ideas from artistic, therapeutic and pedagogical contexts, presenting empirical findings, methodological issues and theoretical considerations relating to dance and disability. The authors' professional backgrounds are as diverse as the topics they chose, ranging from the performing arts to the humanities, education and the cognitive sciences.

The interdisciplinary article by Pini and Maguire-Rosier is presented in an *artistic context*, taking a rather unusual interview form. Through auto-ethnographic analysis, Pini's report sheds light on the transformative importance of the dancing and creatively performing body in coping with the experience of chemotherapy-related cognitive impairment (CRCI), as a form of invisible impairment. In the two contributions by Bläsing and Zimmermann and Fisher, cross-modal translation of artistic processes and performance is presented as an important principle for promoting the participation of people with sensory impairments. Bläsing and Zimmermann address different artistic and technical practices applied to enable persons with visual impairments or blindness to experience dance, and reflect on these practices using the example of an inclusive dance performance by choreographer Simon Mayer. In her exploratory study, Fisher uses embodied song—presented by means of sign language and other forms of bodily expression—as an example to draw attention to the importance of cross-modal transformations focusing on people with hearing impairments. Fisher focuses on the use of poetic metaphors in sign language as well as the transformation of poetry into expressive body movement, both regarded as amodal representations of meaning.

The contributions by Swaine et al. and by Meehan and Carter are set in a *therapeutic context*. Using a quasi-experimental study design, Swaine et al. investigate the added value of a 12-week dance therapy (DT) intervention in combination with usual physical rehabilitation for adults with different physical disabilities, exploring effects on the participants' mobility and participation and their appreciation of DT. Meehan and Carter report on the possibility of treating chronic pain using somatic practices, the main aim of which is to improve the individual's own body and movement perception as well as the perception of the environment, in order to improve self-management in coping with pain. Somatic practices that are typically used as supporting techniques in contemporary dance can convey important insights into the structure and functioning of one's own body and thereby help to improve the awareness, quality, efficiency and creativity of movement through refined perception. The contribution by Meehan and Carter thus enriches the topic of "*Dance and Disability*” with a view to health-promoting dance training and dealing with pain—a common problem among dancers with physical and motor impairments.

As a third perspective, the articles by Bilitza and by Millard et al. address aspects of community dance and education, and thus touch on the *pedagogical context*. In inclusive dance settings, facilitators play an essential role in steering the inclusion process. As part of a qualitative research project, Bilitza examines the views and motivations of professional mediators as moderators promoting inclusion in and through dance. She identifies artistic, social and personal motivation factors that underlie facilitators' engagement in inclusive dance scenarios and substantiate their key role function. Based on the experience gained from two “AllPlayDance” community dance projects, Millard et al. explore the influence of older and more experienced peers in supporting the participation of children with cerebral palsy or autism spectrum disorder, and the role of authorship in creating group dances as a tool for promoting inclusion.

This collection of articles is diverse in terms of focus and demonstrates that there is a wide interest in this field, explored through different methodologies, and viewpoints. At root of many is a deep commitment to enhancing engagement in dance for those with disabilities, for cultural enjoyment or for health and wellbeing. The lived experience of those with disabilities guides many of the articles, pointing to the importance of inclusion as an underlying principle in this field of research.

## Author Contributions

All authors listed have made a substantial, direct, and intellectual contribution to the work and approved it for publication.

## Conflict of Interest

The authors declare that the research was conducted in the absence of any commercial or financial relationships that could be construed as a potential conflict of interest.

## Publisher's Note

All claims expressed in this article are solely those of the authors and do not necessarily represent those of their affiliated organizations, or those of the publisher, the editors and the reviewers. Any product that may be evaluated in this article, or claim that may be made by its manufacturer, is not guaranteed or endorsed by the publisher.

